# Supplementary material for: Oral Cladribine Impairs Intermediate, but Not Conventional, Monocyte Transmigration in Multiple Sclerosis Patients across a Model Blood-Brain Barrier
Source: Int J Mol Sci. 2023 Mar 30;24(7):6487. doi: 10.3390/ijms24076487 (PMC10094666; doi:10.3390/ijms24076487)
Supplement: Supplementary file 1 [file ijms-24-06487-s001.zip › ijms-2268825-supplementary.pdf]

SUPPLEMENTARY MATERIALS

Table S1. Demographic and clinical features of healthy subjects, untreated and cladribine-treated RRMS patients

MS patient ID	Sex (F = 74%)	Age *	Time since MS Diagnosis (months)	Blood Sample at 4-month since Cladribine	Previous Treatment
MS01	F	26	3.5	Yes	-
MS02	F	62	22.0	No	-
MS03	F	53	13.0	No	-
MS04	F	30	0.1	No	-
MS05	F	39	6.4	No	-
MS06	F	69	37.0	No	-
MS07	M	31	0	No	-
MS08	M	47	0	No	-
MS09	M	37	4.5	No	-
MS10	F	39	10.2	Yes	-
MS11	F	41	6.2	Yes	Fingolimod
MS12	F	48	3.5	Yes	-
MS13	M	35	4.2	Yes	-
MS14	F	41	1.8	Yes	Fingolimod
MS15	F	48	21	Yes	Fingolimod
MS16	F	52	18.5	No	Fingolimod
MS17	F	43	8	No	Fingolimod
MS18	M	40	4.2	No	-
MS19	F	41	21	No	Prednisone

Healthy Control (HC) ID	Sex (F = 60%)	Age *
HC01	F	42
HC02	F	28
HC03	F	24
HC04	F	56
HC05	F	30
HC06	F	37
HC07	M	40
HC08	M	43
HC09	M	28
HC10	F	33
HC11	M	24
HC12	M	32
HC13	M	50
HC14	F	43
HC15	F	24
HC16	F	41
HC17	F	35

HC18	F	32
HC19	M	47
HC20	M	52

* Age at which blood samples were taken

F, Female; M, male; MS, Multiple Sclerosis

Table S2 Fluorochrome-conjugated antibodies used for flow cytometric analysis

Cell Marker	Fluorochrome	Clone	Company
**CD3**	Alexa Fluor 532	UCHT1	eBioscience
**CD19**	BV570	HIB19	BioLegend
**CD20**	Super Bright 436	2H7	eBioscience
**CD56**	BV750	5.1H11	BioLegend
**CD14**	Pacific Blue	M5E2	BioLegend
**CD16**	FITC	3G8	Beckman Coulter
**CD38**	BV785	HIT2	BioLegend
**CD45RA**	PerCP-Cy5.5	HI100	BioLegend
**CD197**	PE	G043H7	Beckman Coulter

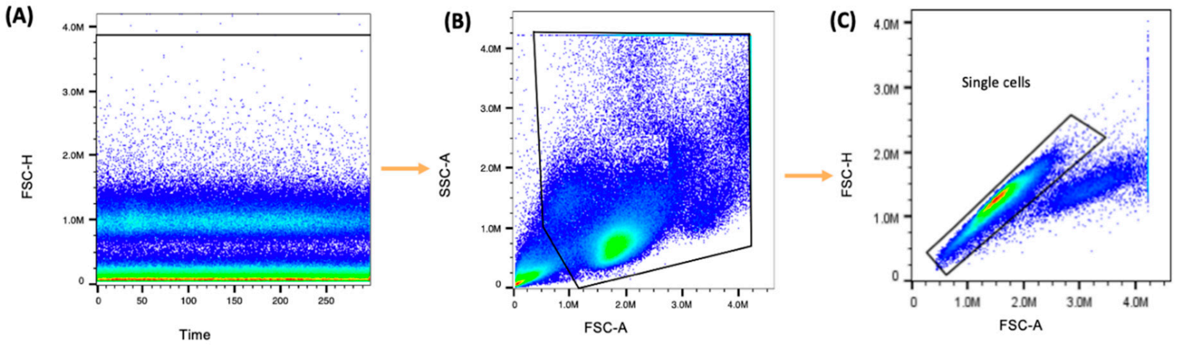

Supplementary Figure S1

Supplementary Figure legends

Supplementary Figure S1. Single cell gating strategy. Cell samples were first gated on time and forward-scatter-height (FSC−H) to ensure data acquisition was consistent over time. Cellular debris and dead cells were removed by gating on side−scatter−area (SSC−A) and forward−scatter−area (FSC−A) to define the total cell population. Doublets and triplets were removed by gating on FSC−A and FSC−H to obtain a single cell population.
